# Trends in surgical procedures for bladder cancer within the Brazilian public health system: An 11-year analysis

**DOI:** 10.14440/bladder.2024.0045

**Published:** 2025-02-25

**Authors:** Heveline R. M. Roesch, Mehrsa Jalalizadeh, Caio de Oliveira, Leonardo O. Reis

**Affiliations:** 1 Department of UroScience, School of Medical Sciences, State University of Campinas, Campinas, São Paulo 13083-872, Brazil; 2 Department of ImmunOncology, School of Life Sciences, Pontifical Catholic University of Campinas, Campinas, São Paulo 13087-571, Brazil

**Keywords:** Bladder cancer epidemiology, Regional disparities, Temporal trends, Surgical treatment

## Abstract

**Background:**

Continuous updates to procedures and hospital admissions for bladder cancer (BC) are crucial for understanding trends, particularly within Brazil’s public health system. Monitoring these data is vital for informed decision-making.

**Objective:**

The objective of the study was to understand the trends in surgical procedures for BC within the Brazilian public health system.

**Methods:**

Data were collected from the Brazilian Data Center for the Public Health System, focusing on hospital admissions related to bladder surgeries from 2013 to 2023. Information was categorized in terms of procedure urgency, hospitalization duration, costs, and mortality rates.

**Results:**

A total of 123,434 BC-related surgical procedures were performed, the majority of which were elective (73.4%) and bladder-preserving (BP, 96.2%). There were 1,710 reported mortalities, with a consistent procedure-specific mortality rate (PSMR) across the 11-year period for all procedures. The average hospitalization duration for elective BP (β = −0.12, *p* < 0.001), elective non-BP (β = −0.46, *p* < 0.001), and urgent non-BP procedures (β = −0.41, *p* = 0.012) steadily decreased. Elective and urgent BPs showed the lowest annual PSMRs (0.66% and 4.25%, respectively), compared to elective and urgent non-BPs (6.93% and 10.72%). The northern and northeastern regions reported significantly fewer cases but higher mortality rates after 2018, despite reduced average hospital stays. While hospital costs for these procedures increased, the standalone costs of surgical interventions remained stable over the 11-year period.

**Conclusion:**

BC-related hospital admissions, particularly for BP procedures, have increased, reflecting improved access to healthcare. However, regional disparities in surgical care, mortality rates, and hospital stays persist across Brazil.

## 1. Introduction

Bladder cancer (BC) represents a significant global health challenge, with varying incidence and mortality rates, influenced by multiple epidemiological and healthcare factors. BC is the 10^th^ most common cancer across the globe. Recent estimates indicated a global incidence of 11 cases/100,000 men and 3.3 cases/100,000 women, with approximately 424,000 new cases being diagnosed annually. In 2020, an estimated 573,000 new cases and 200,000 deaths were reported.^1^ Smoking is the primary risk factor for BC, accounting for 50 – 70% of cases, while occupational exposure to chemicals such as aromatic amines and anilines significantly increases the risk of developing this malignancy,^2^ with potential prognostic implications.^3^

In Brazil, the death rate due to BC in 2017 was notably high, with 3,021 deaths (2.99/100,000) in men and 1,334 deaths (1.29/100,000) in women. By 2020, the incidence rate had increased to 7.12/100,000 in men and 2.61/100,000 in women.^4^

Clinically, BC is suspected when the patient presents with hematuria and is diagnosed through cystoscopy, biopsy, and medical imaging. The disease can be classified as either non-muscle-invasive BC (NMIBC), which accounts for approximately 75% of all cases, or muscle-invasive BC (MIBC) one. The primary treatment for NMIBC is transurethral resection of the bladder tumor (TURBT), whereas radical cystectomy (RC) is performed for MIBC when the tumor has invaded the detrusor muscle.^5^

Brazilian clinicians predominantly follow the American Urological Association (AUA)^6^ and the European Urological Association of Urology (EAU) guidelines.^7^,^8^ These guidelines recommend intravesical immunotherapy with Bacillus Calmette–Guerin for NMIBC following TURBT, typically involving 6 weeks of weekly instillations, with or without maintenance therapy. Cisplatin-based neoadjuvant chemotherapy is recommended for eligible patients before RC, while adjuvant cisplatin-based chemotherapy or immunotherapy is offered to high-risk patients following cystectomy. Both guidelines strongly support bladder-preserving (BP) options due to the high morbidity and significant reduction in quality of life (QoL) associated with RC.^9^,^10^ External beam radiation therapy is administered to selected MIBC patients to facilitate partial cystectomy and avoid the need for RC.

In Brazil, the incidence of BC and related medical procedures exhibit geographic variability, suggesting unique characteristics in local health system management and population demographics that directly impact healthcare access and electronic notification systems. According to Timoteo *et al.*,^11^ recent data indicated an increase in BC-related hospital admissions, consistent with improved access to healthcare services.

Despite its relatively low incidence, BC poses a significant socioeconomic burden, as patients often require multiple elective and urgent procedures, as well as prolonged hospitalization. Furthermore, the inefficient use of health technologies contributes to systemic inefficiencies. The effectiveness of healthcare provision improves when inadequate care is minimized, leading to greater efficiency in delivery and, ultimately, better value for patients.^12^

The Brazilian Unified Health System (SUS) was established following the Federal Constitution of 1988, with the SUS Information Technology Department (DATASUS) created in 1991 to streamline data collection and organization. This infrastructure facilitates the recording and processing of health data from SUS institutions, which are subsequently reported to the Health Assistance Departments of the Ministry of Health.^13^

This article presented data on the procedures involved in BC treatment, including both BP and non-BP approaches. Understanding and updating of these procedures are essential for monitoring trends within the Brazilian public health system.

## 2. Methods

Procedure data were collected in April 2024 from DATASUS, focusing on hospital admissions related to bladder surgical procedures between 2013 and 2023. The data included procedure urgency, length of hospitalization, associated costs, and mortality rates.

The DATASUS comprises several key systems and databases, including the National Register of Health Establishments, the SUS Outpatient Information System, and the SUS Hospital Information System. These systems are designed to register all healthcare services resulting from hospital admissions and are funded by SUS, utilizing data captured from Hospital Admission Authorization forms.^14^

The procedures were categorized into two groups: BP and non-BP procedures. BP procedures included bladder tumor endoscopic resection, oncological bladder tumor endoscopic resection, and partial cystectomy (codes 409010383, 0416010172, and 0409010022). In contrast, non-BP procedures involved RC, single-stage RC with urinary diversion, oncological single-stage RC with urinary diversion, and oncological RC with urinary diversion (codes 0409010030, 0409010049, 0416010024, and 0416010032). A total of three BP procedures and four non-BP were included in the analysis.

### 2.1. Statistical analysis

The data are presented as sums, means, and standard deviations (SD). Temporal trends were analyzed using two methods. The first method employed the Surveillance, Epidemiology, and End Results Program of the National Cancer Institute to analyze the number of procedures and mortalities. The second method involved a simple linear regression, applied to other variables, including hospitalization duration, costs, and mortality rates, which contained zero values, such as the procedure-specific mortality rate (PSMR). The results of the simple linear regression model were considered significant if the *p* < 0.05.

### 2.2. The surveillance, epidemiology, and end results program method

Initially, a joinpoint analysis was performed using the R package “segmented.” The annual percentage changes (APCs) were calculated by taking the exponent of the slopes obtained from a logarithmic transformation of the data. The average APCs (AAPCs) were computed based on the weight of each segment. To address the limitations of traditional methods for small sample sizes, a Bayesian approach was used to calculate confidence intervals (CI) for APCs and AAPCs. This robust method incorporated prior information and probabilistic inference. The Bayesian model was coded using the “stan” language package in R, and the parameters were adjusted to reduce divergence in the model. APCs and AAPCs were considered significant if the CI excluded the zero. All analyses were performed using R version 4.3.3 (Austria) and RStudio (version 2024.04.2+764; Austria).

## 3. Results

**Figure 1:**
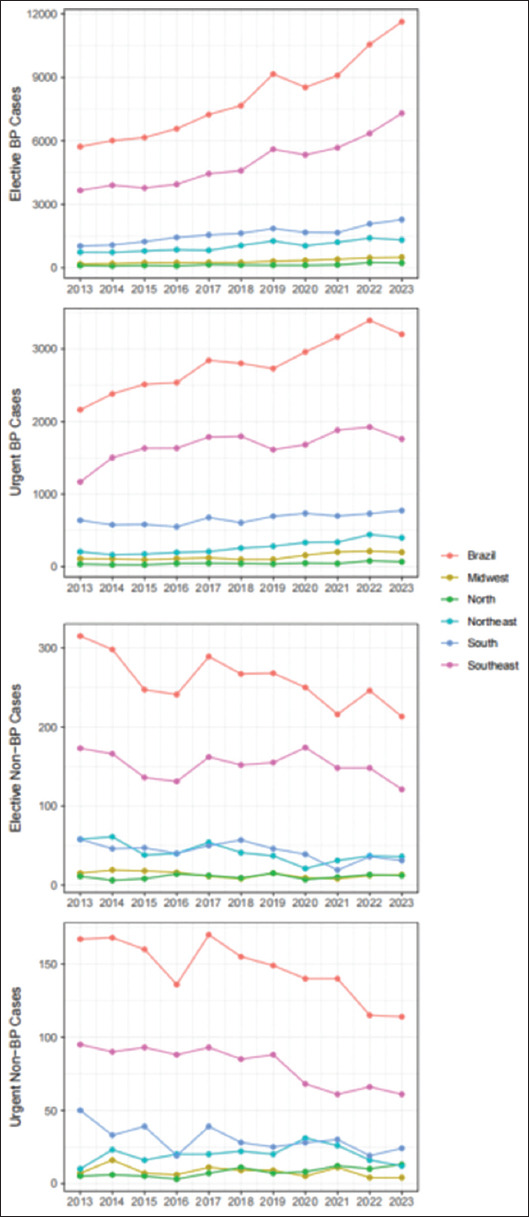
Number of procedures performed annually by region Abbreviation: BP: Bladder-preserving.

Throughout the 11-year period, a total of 123,434 BC-related procedures were performed, the majority of which were elective (73.4%) and BP (96.2%). The highest number of procedures per 10,000 population occurred in the south (11.5), while the lowest was in the north (1.3) (Table 1). The number of procedures steadily increased throughout the period, with no joinpoints observed (AAPC = 6.2, 95% CI = 5.4 – 7.0). The increase in BP procedures was the primary driver behind this trend. Both elective and urgent BP procedures rose across all five regions of Brazil (Brazil AAPC = 7.4 and 4.0, respectively), while elective and urgent non-BP procedures decreased or remained stable, respectively (Brazil AAPC = −2.6 and −3.5), as shown in Figure 1 and Table 1.

**Table 1:** Key statistics pertaining to bladder cancer procedures from 2013 to 2023

Parameter	Brazil	North	Northeast	Southeast	South	Midwest
Population	203,062,512	17,349,619	54,644,582	84,847,187	29,933,315	16,287,809
Procedures						
All						
Total, no.	123,434	2,269	14,936	75,525	25,599	5,105
Mean annual±SD, no.	11,221±2,241	206±71	1,358±328	6,866±1305	2,327±437	464±142
AAPC (95% CI)	6.2 (5.4 – 7.0)	8.6 (4.9 – 12.6)	7.2 (5.5 – 9.4)	5.7 (4.9 – 6.5)	5.6 (4.0 – 7.1)	8.9 (6.3 – 11.6)
Per 10,000 population, no. (mean annual±SD)	6.1 (0.55±0.11)	1.3 (0.12±0.04)	2.7 (0.25±0.06)	8.9 (0.81±0.15)	8.6 (0.78±0.15)	3.1 (0.28±0.09)
Elective BP procedures						
Total no. (mean annual±SD)	88,333 (8,030.3±1943.6)	1,560 (141.8±53.0)	11,261 (1,023.7±246.9)	54,585 (4,962.3±1,188.3)	17,526 (1,593.3±388.7)	3,401 (309.2±108.6)
AAPC (95% CI)	7.4 (6.4 – 8.8)	8.4 (3.1 – 13.2)	7.2 (5.0 – 9.5)	7.1 (5.8 – 8.5)	7.3 (5.9 – 8.9)	10.5 (7.7 – 12.4)
Urgent BP procedures						
Total no. (mean annual±SD)	30,633 (2,784.8±369.47)	505 (45.9±16.0)	3,004 (273.1±94.4)	18,384 (1,671.3±207.9)	7,269 (660.8±73.9)	1,471 (133.7±39.2)
AAPC (95% CI)	4.0 (3.0 – 5.1)	8.8 (4.3 – 14.6)	10.4 (7.2 – 12.4)	2.6 (0.1 – 4.5)	2.8 (1.0 – 4.2)	7.0 (3.4 – 11.1)
Elective non-BP procedures						
Total no. (mean annual±SD)	2,850 (259.1±32.2)	117 (10.6±2.9)	454 (41.3±11.9)	1,666 (151.5±17.1)	469 (42.6±11.4)	144 (13.1±3.9)
AAPC (95% CI)	−2.6 (−4.4 – −0.4)	2.5 (−3.5 – 8.3)	−5.4 (−9.7 – −0.7)	−1.0 (−3.8 – 1.7)	−6.4 (−10.2 – −2.6)	−4.2 (−8.7 – 1.1)
Urgent non-BP procedures						
Total no. (mean annual±SD)	1,614 (146.7±19.8)	87 (7.9±3.2)	216 (19.6±6.0)	888 (80.7±13.7)	334 (30.4±9.4)	89 (8.1±3.6)
AAPC (95% CI)	−3.5 (−5.1 – −1.4)	11.1 (3.3 – 18.4)	2.0 (−6.0 – 10.1)	−4.9 (−6.8 – −3.1)	−6.0 (−10.3 – −1.3)	−6.2 (−14.2 – 2.6)
Proportion elective						
Mean annual±SD, %	73.4±2.4	73.8±2.9	78.7±2.4	74.0±3.4	69.7±4.0	69.0±3.6
β (*p*−value)	0.006 (0.002)	−0.000 (0.86)	−0.005 (0.024)	0.007 (0.017)	0.010 (0.004)	0.007 (0.033)
Proportion BP						
Mean annual±SD, %	96.2±1.1	91.7±1.6	95.1±1.9	96.5±0.9	96.7±1.3	94.9±2.4
β (*p*-value)	0.003 (<0.001)	0.002 (0.26)	0.005 (<0.001)	0.003 (<0.001)	0.006 (<0.001)	0.006 (<0.001)
Mortality						
All						
Total, no.	1,710	46	256	1,016	325	67
Mean annual, no.±SD	155±25	4±2	23±6	92±12	30±8	6±3
AAPC (95% CI)	3.2 (−0.3 – 6.0)	6.5 (−8.1 – 21.1)	−3.1 (−10.3 – 4.1)	2.7 (0.5 – 4.7)	5.9 (2.3 – 10.1)	9.4 (−5.4 – 25.5)
APC 2013 – 2018 (95% CI)	-	−14.1 (−44.0 – 6.6)	−14.3 (−22.2 – −7.5)	-	-	-
APC 2019 – 2023 (95% CI)	-	31.3 (21.3 – 42.3)	−7.9 (−30.7 – 7.6)	-	-	-
PSMR: Elective BP						
Mean annual±SD, %	0.66±0.60	1.16±3.52	1.31±2.96	0.83±1.03	0.16±0.17	0.15±0.28
β (*p*-value)	0.0002 (0.57)	0.0037 (0.13)	0.0013 (0.54)	−0.0006 (0.42)	−0.0000 (0.85)	0.0002 (0.28)
PSMR: Urgent BP						
Mean annual±SD, %	4.25±2.21	7.96±15.41	4.81±4.54	5.01±3.55	3.73±3.07	2.99±7.07
β (*p*-value)	0.0007 (0.65)	0.0133 (0.21)	−0.0053 (0.081)	0.0023 (0.34)	0.0034 (0.10)	−0.0074 (0.12)
PSMR: Elective non-BP						
Mean annual±SD, %	6.93±1.79	6.14±6.48	7.68±4.25	7.26±2.63	7.26±4.86	2.27±4.09
β (*p*-value)	0.0006 (0.76)	−0.0164 (0.001)	−0.0041 (0.33)	0.0012 (0.65)	0.0081 (0.080)	0.0035 (0.39)
PSMR: urgent non-BP						
Mean annual±SD, %	10.72±2.21	5.50±8.67	11.68±11.49	11.20±3.10	10.45±5.17	11.52±10.46
β (*p*-value)	0.0029 (0.18)	0.0073 (0.41)	−0.0099 (0.40)	0.0094 (0.049)	0.0013 (0.68)	0.0042 (0.69)
Days hospitalized						
All procedures						
Total, days	482,472	13,334	64,642	283,986	97,719	22,791
Mean annual±SD, days	43,861±3,174	1,212±379	5,877±942	25,817±1,625	8,884±793	2,072±371
β (*p*-value)	661.9 (0.018)	76.7 (0.023)	240.0 (0.001)	320.9 (0.029)	−6.2 (0.94)	30.5 (0.42)
Elective BP procedures						
Mean annual±SD, days	21,192±2,318	633±229	3,233±499	12,718±1,388	3,799±502	808±138
Average per procedure±SD, days	2.7±0.4	4.6±0.9	3.2±0.5	2.6±0.4	2.5±0.5	2.8±0.7
β (*p*-value)	−0.12 (<0.001)	−0.16 (0.064)	−0.12 (<0.001)	−0.11 (<0.001)	−0.14 (<0.001)	−0.18 (<0.001)
Urgent BP procedures						
Mean annual±SD, days	17,236±1,879	347±185	1,992±619	9,797±1,091	4,162±378	938±259
Average per procedure±SD, days	6.2±0.5	7.3±1.8	7.4±0.9	5.9±0.4	6.3±0.7	7.1±1.5
β (*p*-value)	−0.08 (0.060)	0.21 (0.23)	−0.06 (0.48)	−0.07 (0.075)	−0.16 (0.010)	−0.19 (0.20)
Elective non-BP procedures						
Mean annual±SD, days	3,380±733	146±62	439±164	2,097±421	516±199	183±94
Average per procedure±SD, days	12.9±1.7	13.8±3.8	10.5±1.2	13.8±2.2	11.7±2.0	13.3±4.9
β (*p*-value)	−0.46 (<0.001)	−0.65 (0.071)	−0.24 (0.036)	−0.57 (<0.001)	−0.35 (0.071)	−0.73 (0.13)
Urgent non-BP procedures						
Mean annual±SD, days	2,054±454	86±36	212±71	1,206±289	407±169	143±94
Average per procedure±SD, days	13.9±1.9	11.6±6.5	11.2±3.8	14.8±2.0	13.1±2.8	17.4±6.5
β (*p*-value)	−0.41 (0.012)	−0.64 (0.32)	−0.23 (0.56)	−0.21 (0.30)	−0.46 (0.079)	−1.41 (0.013)
Cost						
All procedures						
Total cost, R$	171,204,463	3,430,834	21,427,018	104,031,028	35,457,102	6,858,481
Mean annual cost±SD, R$	15,564,042±3,127,040	311,894±115,968	1,947,911±459,277	9,457,366±1,806,218	3,223,373±616,004	623,498±195,910
β (*p*-value)	921,746 (<0.001)	30,260 (<0.001)	132,817 (<0.001)	530,689 (<0.001)	174,347 (<0.001)	53,633 (<0.001)
Average procedure cost±SD, R$	1,387±16	1,504±103	1,437±61	1,377±20	1,385±34	1,342±52
β (*p*-value)	0.68 (0.68)	18.60 (0.052)	−0.31 (0.96)	0.41 (0.084)	0.00 (0.99)	−0.84 (0.88)
Elective BP procedures						
Mean annual cost±SD, R$	9,118,182±2,568,417	156,225±74,681	1,200,594±341,122	5,609,425±1,571,684	1,821,119±491,600	330,819±145,960
Average procedure cost±SD, R$	1,126±47	1,077±100	1,162±66	1,121±47	1,136±39	1,042±97
β (*p*-value)	14.0 (<0.001)	18.8 (0.039)	16.5 (0.002)	13.5 (<0.001)	11.0 (<0.001)	26.8 (<0.001)
Urgent BP procedures						
Mean annual cost±SD, R$	3,786,508±777,462	56,185±24,211	388,698±154,227	2,256,013±417,005	914,014±171,670	171,597±70,585
Average procedure cost±SD, R$	1,348±109	1,197±170	1,401±184	1,341±107	1,374±119	1,250±197
β (*p*-value)	30.8 (<0.001)	38.5 (0.008)	34.3 (0.043)	29.2 (<0.001)	32.6 (<0.001)	41.7 (0.016)
Elective non-BP procedures						
Mean annual cost±SD, R$	1,746,888±200,711	62,066±12,843	261,132±65,512	1,061,326±116,261	285,233±75,586	77,131±25,090
Average procedure cost±SD, R$	6,752±228	6,016±1,124	6,436±691	7,015±244	6,712±666	5,851±800
β (*p*-value)	33.7 (0.13)	37.6 (0.74)	51.9 (0.46)	−0.2 (0.99)	135.3 (0.023)	−3.0 (0.97)
Urgent non-BP procedures						
Mean annual cost±SD, R$	912,465±82,393	37,418±18,018	97,486±32,858	530,602±75,700	203,007±48,973	43,951±16,884
Average procedure cost±SD, R$	6,263±435	4,669±1182	5,047±1134	6,606±283	6,840±1144	5,588±962
β (*p*-value)	99.4 (0.006)	96.0 (0.42)	200.3 (0.057)	68.9 (0.003)	226.3 (0.028)	35.5 (0.72)

Note: β represents the coefficient or slope of the line in the regression analysis of annual changes. The value of β indicates the unit change per year. Abbreviations: AAPC: Average annual percent change; APC: Annual percent change; BP: Bladder preserving; CI: Confidence interval; no.: Number; PSMR: Procedure-specific mortality rate; R$: Brazilian real; SD: Standard deviation.

### 3.1. Mortality

A total of 1,710 mortalities were reported for these procedures. The annual mortality rate steadily rose without joinpoints (AAPC = 4.7, 95% CI = 0.5 – 8.6). In the regional analysis of mortality, a joinpoint was detected in the north and northeast regions in 2018, with a negative APC before and a positive APC after this year (Table 1).

**Figure 2:**
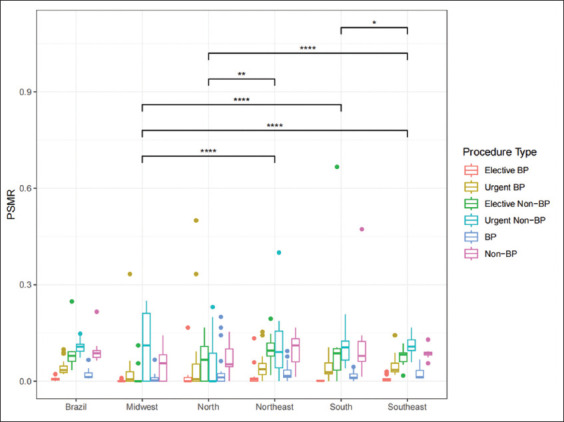
Comparison of PSMR across regions using the Wilcoxon rank-sum test. Notes: The symbols *, **, ***, and **** correspond to significance levels of 0.05, 0.01, 0.001, and 0.0001, respectively. Abbreviations: BP: Bladder-preserving; PSMR: Procedure-specific mortality rate.

The mean annual PSMR varied among the procedures, with the lowest rates observed for elective and urgent BP procedures (0.66% and 4.25%, respectively), and the highest rates for elective and urgent non-BP procedures (6.93% and 10.72%, respectively). The PSMR remained steady throughout the 11-year period for all the procedures. Figure 2 illustrates PSMR comparisons across regions.

### 3.2. Hospitalization duration

A total of 482,472 days of hospitalization were reported for BC-related procedures. A positive annual trend in hospitalization days was observed (β = 661.9, *p* = 0.018), and was expected to be ascribed to the increased number of procedures. Conversely, the average hospitalization duration per procedure steadily dropped over the study period, with a downward slope detected for elective BP (β = −0.12, *p* < 0.001), elective non-BP (β = −0.46, *p* < 0.001), and urgent non-BP procedures (β = −0.41, *p* = 0.012).

**Figure 3:**
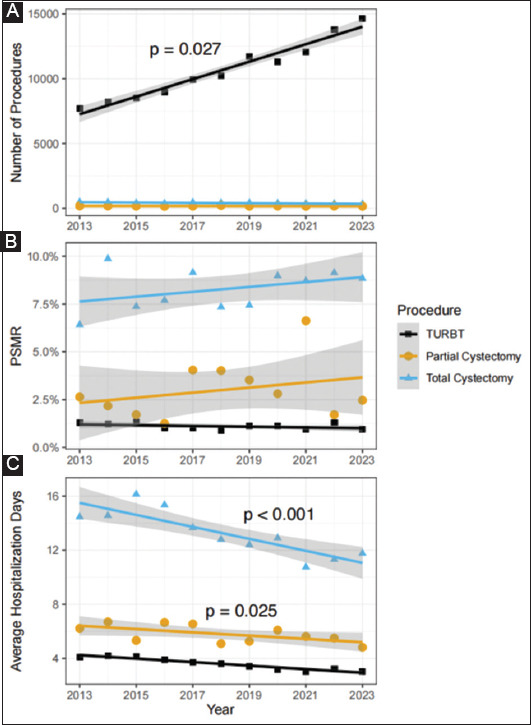
(A-C) Temporal trends in TURBT, partial cystectomy, and total cystectomy procedures in the country. Elective and urgent procedures are not separated. *p*-values were obtained using simple linear regression, and only significant values are shown. Abbreviations: PSMR: Procedure-specific mortality rate; TURBT: Transurethral resection of bladder tumor.

To assess whether the decline in the average hospitalization days in the BP category was due to an increase in the number of TURBT, which required shorter hospitalization duration (3.6 ± 0.5 days), we compared them to partial cystectomy (5.8 ± 0.7 days). Two factors were found to contribute to this decline. First, the proportion of TURBT procedures in the BP category increased from 97.6% in 2013 to 98.9% in 2023 (β = 0.0012, *p* < 0.001). Second, the average length of hospitalization for elective TURBT procedures steadily decreased. In 2013, elective TURBT patients spent an average of 3.31 days in the hospital, compared to 2.21 days in 2023 (β = −0.12, *p* < 0.001). Figure 3 shows the temporal trends for these procedures in the country. Elective and urgent procedures are not separated, and only significant *p*-values are presented.

### 3.3. Costs

**Figure 4:**
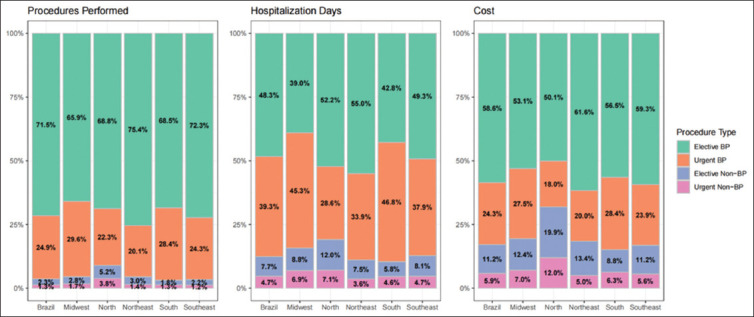
Proportional comparison of procedure types and their associated healthcare expenses by region Abbreviation: BP: Bladder-preserving.

A total of 171,204,463 Brazilian reais (R$) were spent on BC-related procedures in the country between 2013 and 2023. The average cost per procedure was R$1,387 ± 16, which remained stable over the 11-year period (β = 14.0, *p* < 0.001). However, the average cost increased for elective BP, urgent BP, and urgent non-BP procedures (β = R$14.0, R$30.8, and R$99.4 per year, respectively; *p* < 0.007 for all; Table 1). Figure 4 illustrates the proportional comparison of procedures performed, hospitalization days, and costs across regions.

## 4. Discussion

This study provided a comprehensive overview of BC in Brazil over the past decade, facilitating comparisons with existing data and highlighting trends that offer valuable insights. DATASUS is one of the largest and most comprehensive healthcare databases in the world, and the trends observed in this dataset likely mirror the genuine impact of public health policies, including those targeting genitourinary cancers.^15^

We employed APC and AAPC to analyze the trends, both being widely recognized methods for describing cancer statistics.^16^ These methodologies offer a standardized approach, allowing for comparisons across different populations and cancer types. We analyzed seven bladder surgical procedures, categorizing them into BP and non-BP groups.

The 2019 epidemiological study by Timoteo *et al*.^11^ is particularly relevant to our analysis. They categorized BC surgical procedures into “open surgery” (including cystectomies and cystoenteroplasty) and “endoscopic surgery” (comprising two types of endoscopic bladder resections). Our analysis of recent data on BC and its clinical management reveals both similarities and differences when compared with their findings from 2008 to 2017.

During our 11-year study period, we recorded a total of 123,434 BC-related procedures, with a conspicuous increase in the number of interventions, particularly BP procedures. This growth was most pronounced in the southern regions of Brazil, where the procedural rate per 10,000 population was up to 11.5. This increase in reported procedures aligns with previous data, which documented a rise in admissions from 7,277 in 2008 to 16,547 in 2017.^11^ This upward trend has been partially attributed to improved access to healthcare and more accurate reporting.

The relationship between BP and non-BP procedures is particularly noteworthy in our findings. Both elective and urgent BP procedures increased, whereas non-BP procedures either decreased or remained stable. In recent years, BP procedures have accounted for the majority of BC hospital treatments in Brazil, particularly in the south. These patterns indicate a shift in clinical practice toward less invasive interventions, reflecting a growing preference for conservative treatment strategies. Timoteo *et al*.^11^ also reported a decrease in the C/T ratio (the correlation between RC and TURBT) for BC treatment, from 0.19 in 2008 to 0.08 in 2017. This decrease suggests improvements in the early diagnosis and treatment of NMIBC.

As previously mentioned, both the AUA and EAU guidelines recommend bladder preservation due to the significant reduction in QoL associated with RC. The observed decline in non-BP procedures in our data may be attributed to these recommendations. A study from Germany reported no change in the rate of RC between 2006 and 2017,^17^ whereas two studies from the United States identified a declining trend in the procedure.^18^,^19^ Unfortunately, both studies used data harvested before 2013, and we were unable to find recent studies on these trends.

A 2020 study from Scotland reported improved efficacy of TURBT following the introduction of mandatory quality indicators (QI) in 2014. These QIs included single post-TURBT instillation of mitomycin C, improved resection technique, and early re-TURBT for certain high-risk patients.^20^ The most recent AUA and EAU guidelines also recommend these QIs. Their introduction into Brazil may explain the rise in TURBT procedures (due to re-TURBTs) and the decline in partial cystectomies (likely due to a reduced recurrence rate).

With regard to mortality, our data indicated a lower PSMR for BP procedures, with a rate of 0.66% for elective cases and a rate of 4.25% for urgent cases. A previous report gave similar rates for elective TURBT (0.6%) and urgent TURBT (2.6%),^11^ whereas the mortality rates for non-BP procedures were significantly higher. It is important to note that mortality rates for urgent radical cystectomies are approximately 50% higher than those for elective procedures,^21^,^22^ underscoring the greater severity of cases managed in urgent settings. This suggests that, despite advancements in BP, managing more advanced cases continues to present considerable challenges.

Brazil is a vast country characterized by significant socioeconomic heterogeneity.^15^ A landmark 2021 study by Fonseca *et al*.^23^ revealed that cancer patients in some regions had to travel hundreds of kilometers to access definitive treatment. These regional disparities are reflected in our data. For instance, although the northeast had more than double the population of the south, fewer procedures were performed there, suggesting that patients often migrate to other regions for care. Our previous epidemiological study highlighted higher per capita health expenditures, a greater number of government-funded hospital beds, and a higher prevalence of robotic surgical units in the south and southeast regions.^15^ Our data also revealed that certain regions performed more emergency surgeries than others, leading to higher service costs due to increased hospitalization days. This disparity might have stemmed from deficiencies in elective care, which remains unbalanced in some areas.

Concerning hospitalization duration, our findings revealed a total of 482,472 days of hospitalization, with a downward trend in the average length of stay per procedure over time, particularly for elective and non-BP procedures. The average length of stay for TURBT was 3.6 ± 0.5 days, compared to 5.8 ± 0.7 days for partial cystectomy. However, Timoteo *et al*.^11^ reported slightly higher averages: 4.3 days for TURBT, 6.9 days for partial cystectomies, and 13.6 days for radical cystectomies. The reduction in hospitalization duration may suggest an increased effectiveness of minimally invasive interventions and improved post-operative recovery, reflecting an evolution in therapeutic approaches.

Unfortunately, no data are available on the costs of BC-related procedures that would allow for a comparison between the two periods. The overall cost of procedures recorded in DATASUS reflects the broader hospital context rather than the specific costs of individual procedures, which may remain stable due to the government’s established pricing tables. Despite the stability of procedural costs over the past decade, expenses related to hospital admissions have increased. This phenomenon may be attributed to the costs associated with protracted hospital stays in cases of late-stage diagnosis and advanced complications, particularly in low-income regions.

Our findings demonstrated slightly positive trends in BC over the past ten years. The detailed reporting of BC statistics in Table 1 is a strength of our study, as it updates previous reports on BC in the Brazilian population and offers insights that can inform the design of public health policies. The comprehensive regional and temporal analyses presented in this table provide valuable data for epidemiological and economic studies. It is crucial to recognize the variability in healthcare access across different regions of Brazil, as evidenced by the differences in surgical techniques employed for similar conditions. Addressing these regional disparities may necessitate tailored financial and policy approaches to ensure equitable quality of care across the country.

Our study has several limitations. As an epidemiological investigation, our findings do not lead to definitive conclusions. Like other neoplasms, BC may be under-reported as a cause of hospitalization and death, particularly in developing countries such as Brazil. Within the DATASUS registration system, procedures are not exclusively designed for BC treatment, making it impossible to filter searches specifically for malignant bladder neoplasms (ICD-10 code C67). Consequently, although most surgical records were related to BC, some might include procedures for other conditions.

Despite the limitations of the DATASUS platform, such as data gaps, reliability, and accuracy concerns, as well as challenges in integration, it remains a valuable resource for understanding the dynamics of the public healthcare system. Furthermore, DATASUS does not capture procedures performed using private resources, reinforcing its role as the primary source of unified data for the Brazilian public health system, which serves approximately 75% of the population.^24^

## 5. Conclusion

Hospital admissions related to BC, particularly those involving BP procedures, have been on the rise, reflecting improved access to healthcare services within the public system. However, significant regional disparities persist in surgical care, mortality rates, and hospital stays across different regions of Brazil. While the hospital costs associated with these procedures have risen, the costs of individual surgical interventions have remained stable over the past 11 years.

## Data Availability

The datasets analyzed are available from the corresponding author upon reasonable request.
